# Supplementary material for: A Study of the Community Relationships Between Methanotrophs and Their Satellites Using Constraint-Based Modeling Approach
Source: Int J Mol Sci. 2024 Nov 20;25(22):12469. doi: 10.3390/ijms252212469 (PMC11594979; doi:10.3390/ijms252212469)
Supplement: Supplementary file 1 [file ijms-25-12469-s001.zip › ijms-3280422-supplementary.pdf]

Supplementary Material

Supplementary Figures and Tables

Supplementary Figures

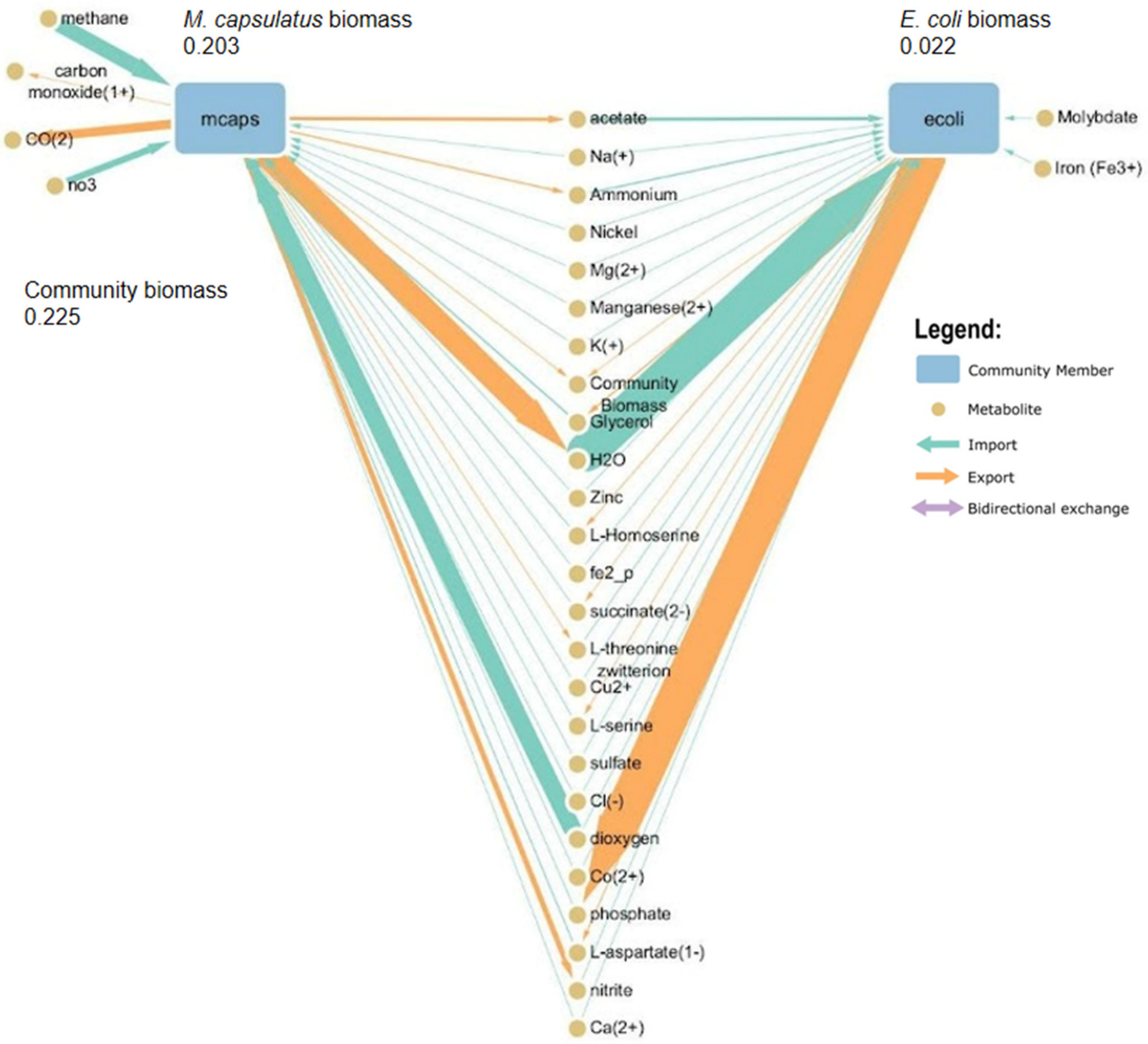

Supplementary Figure S1. Visualization of cross-feeding metabolites in the *i*McBath and *i*EC1372_W3110 community under oxygen-limited conditions using ScyNet. The color shows which metabolites are consumed (turquoise lines) and which are excreted (orange lines) by community members from the environment. The width of the line designates the flux value through this reaction.

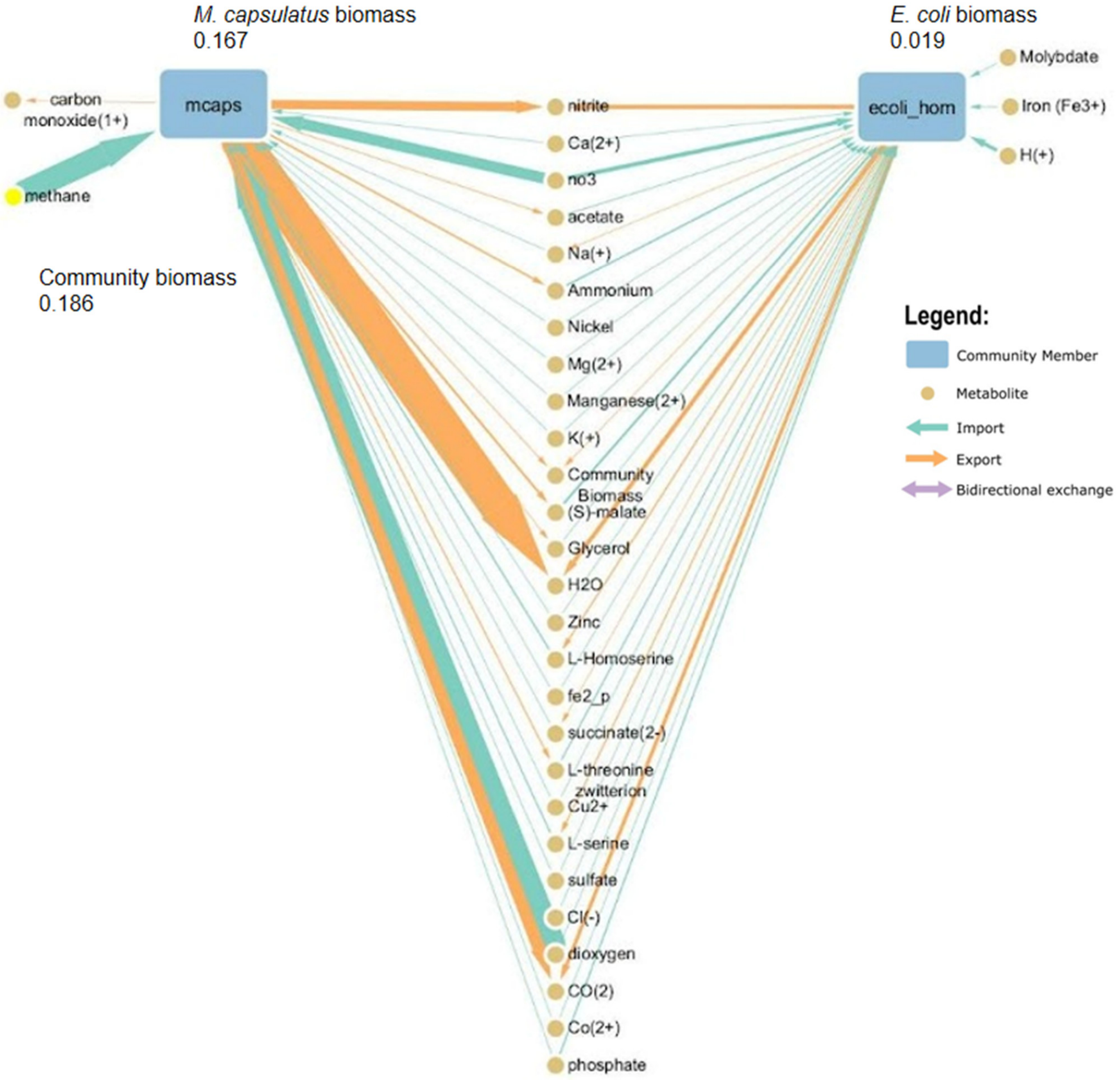

Supplementary Figure S2. Visualization of cross-feeding metabolites in the *i*McBath and modified *i*EC1372_W3110 community under oxygen-limited conditions using ScyNet.The color shows which metabolites are consumed (turquoise lines) and which are excreted (orange lines) by community members from the environment. The width of the line designates the flux value through this reaction.

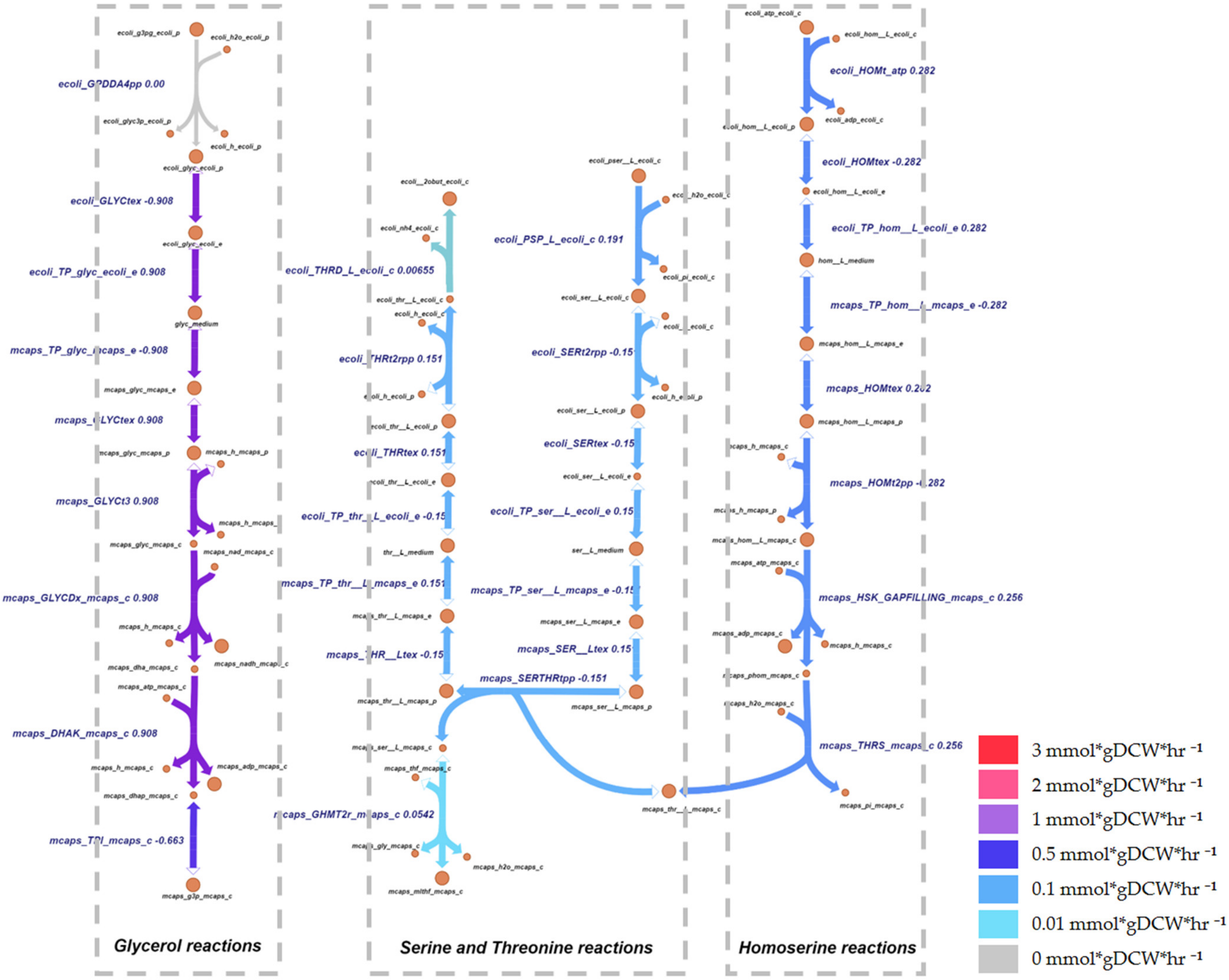

Supplementary Figure S3. Visualization with Escher of cross-feeding of glycerol and amino acids in the community model of *i*McBath and *i*EC1372_W3110 under oxygen-limited conditions. The legend in the figure indicates the flux intensity designations, and dashed lines represent reactions related to the production, exchange, and assimilation of glycerol, serine, threonine, and homoserine.

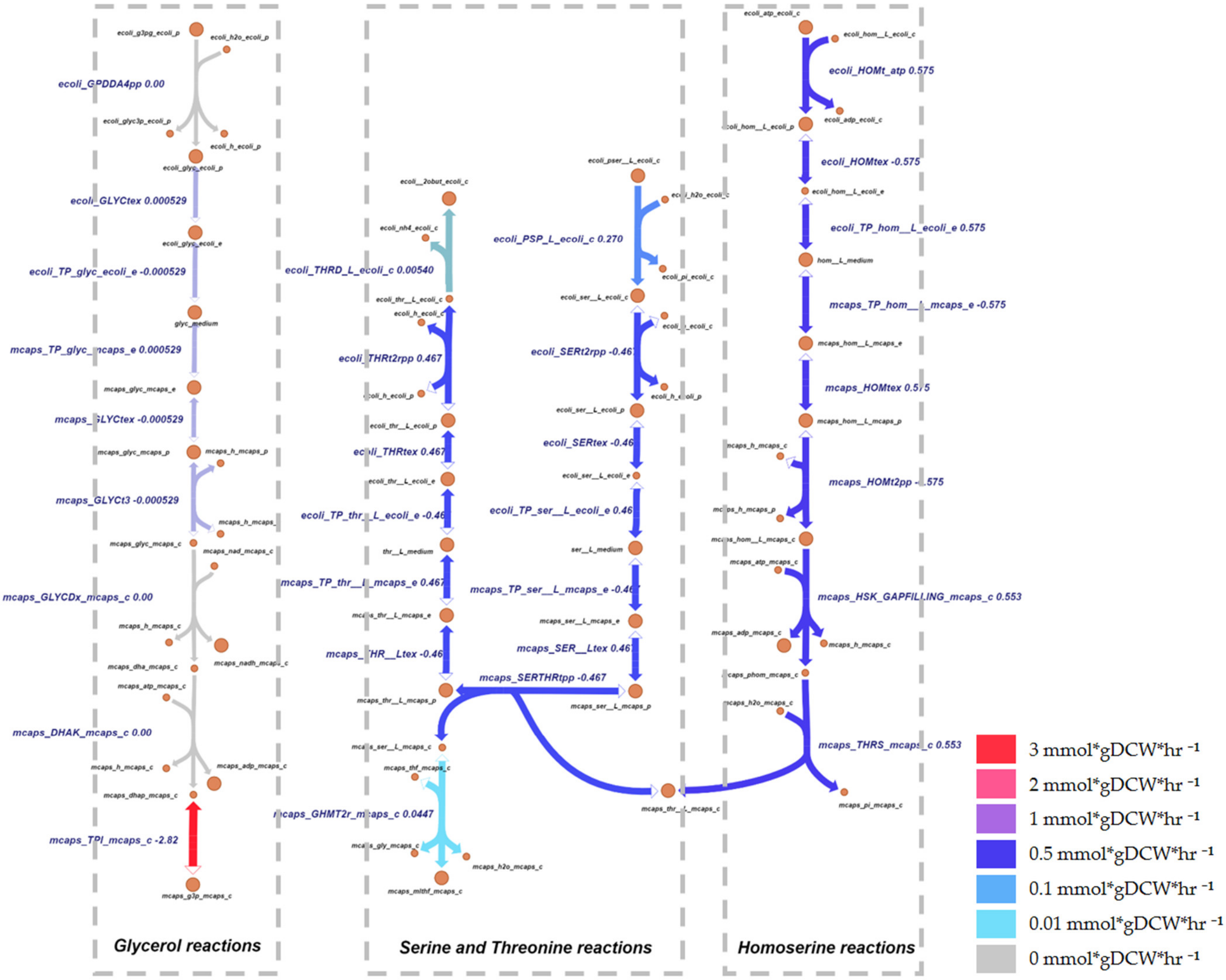

Supplementary Figure S4. Visualization with Escher of cross-feeding of glycerol and amino acids in the community model of *i*McBath and modified *i*EC1372_W3110 under oxygen-limited conditions. The legend in the figure indicates the flux intensity designations, and dashed lines represent reactions related to the production, exchange, and assimilation of glycerol, serine, threonine, and homoserine.

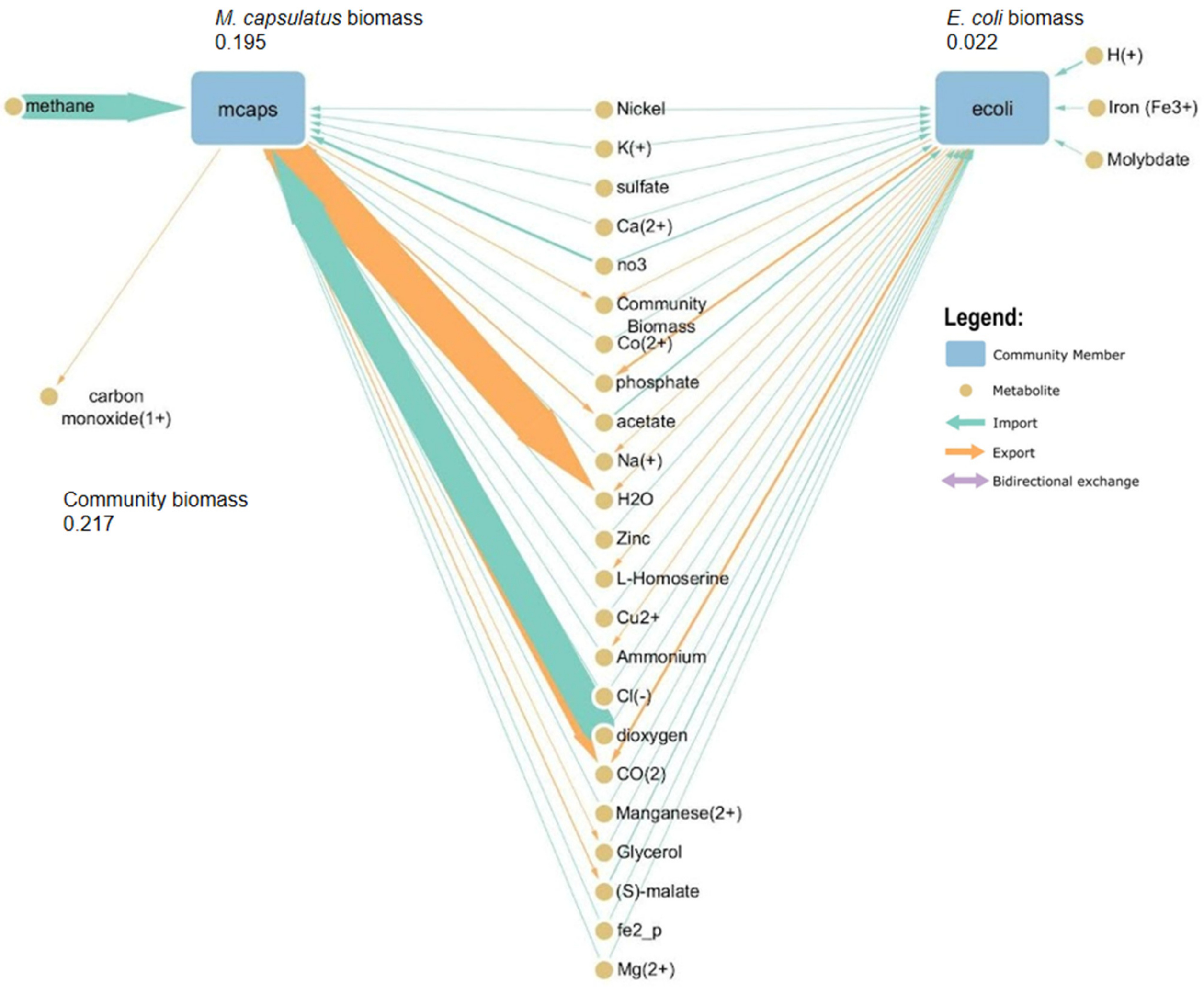

Supplementary Figure S5. Visualization of cross-feeding metabolites in the *i*McBath and *i*EC1372_W3110 community under nitrogen-limited conditions using ScyNet.The color shows which metabolites are consumed (turquoise lines) and which are excreted (orange lines) by community members from the environment. The width of the line designates the flux value through this reaction.

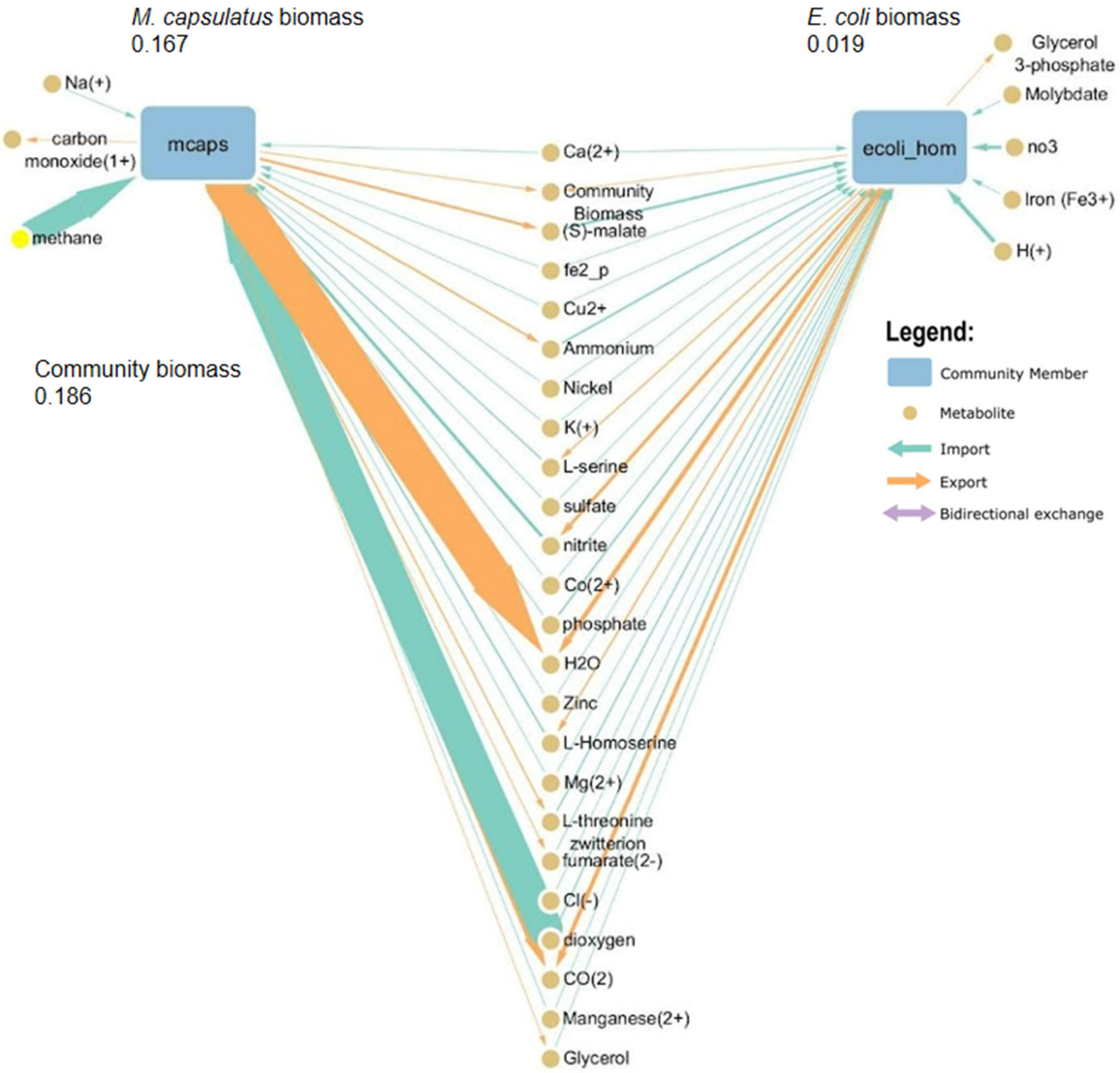

Supplementary Figure S6. Visualization of cross-feeding metabolites in the *i*McBath and modified *i*EC1372_W3110 community under nitrogen-limited conditions using ScyNet.The color shows which metabolites are consumed (turquoise lines) and which are excreted (orange lines) by community members from the environment. The width of the line designates the flux value through this reaction.

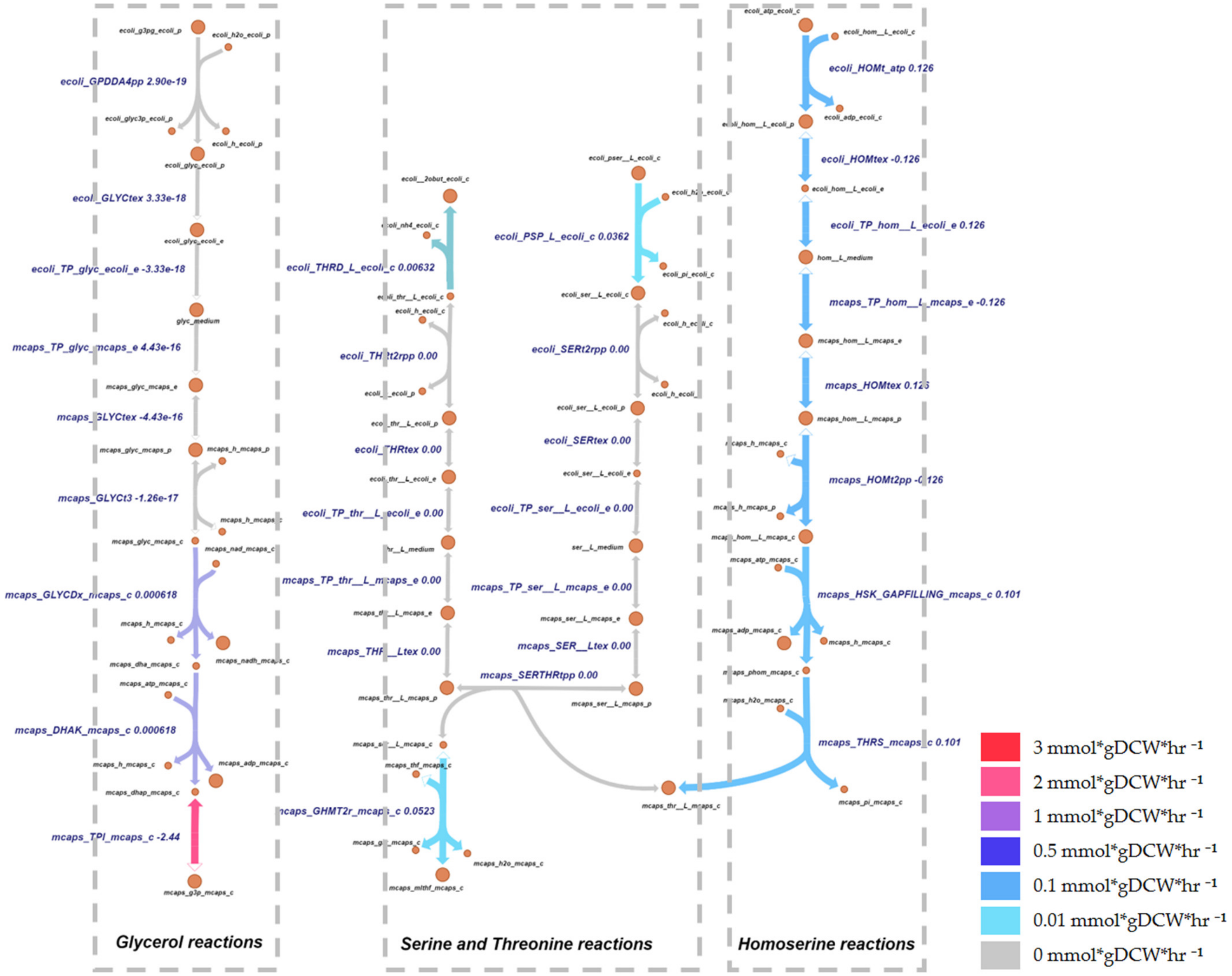

Supplementary Figure S7. Visualization with Escher of cross-feeding of glycerol and amino acids in the community model of *i*McBath and *i*EC1372_W3110 under nitrate-limited conditions. The legend in the figure indicates the flux intensity designations, and dashed lines represent reactions related to the production, exchange, and assimilation of glycerol, serine, threonine, and homoserine.

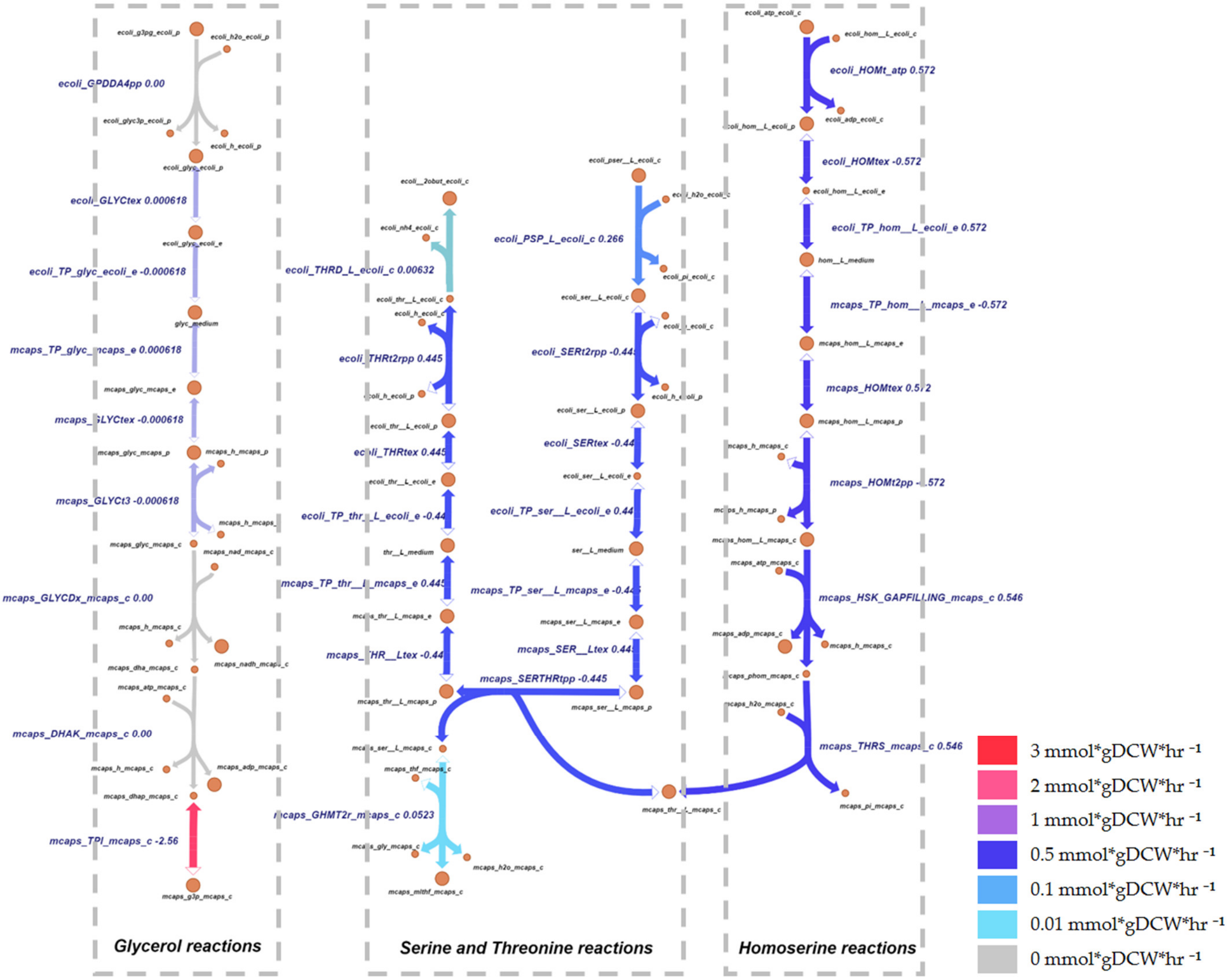

Supplementary Figure S8. Visualization with Escher of cross-feeding of glycerol and amino acids in the community model of *i*McBath and modified *i*EC1372_W3110 under nitrate-limited conditions. The legend in the figure indicates the flux intensity designations, and dashed lines represent reactions related to the production, exchange, and assimilation of glycerol, serine, threonine, and homoserine.

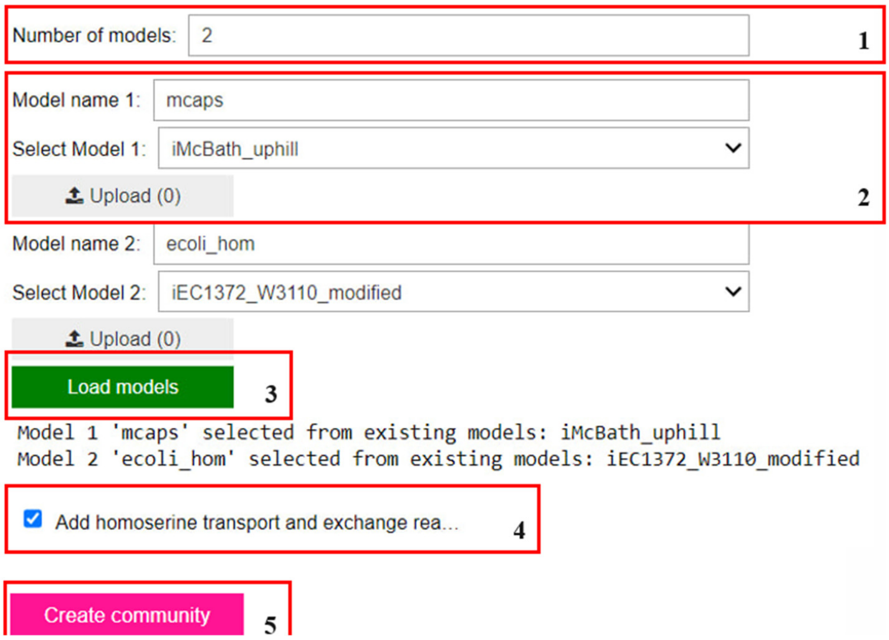

Supplementary Figure S9. The model loading step includes: **1** Selection of the number of models to be used for community reconstruction; **2** Loading or selecting models used in the study; **3** A button for loading models; **4** Checkbox for adding transport and exchange reactions for homoserine in the *i*McBath_uphill model; **5** A button for the community model reconstruction.

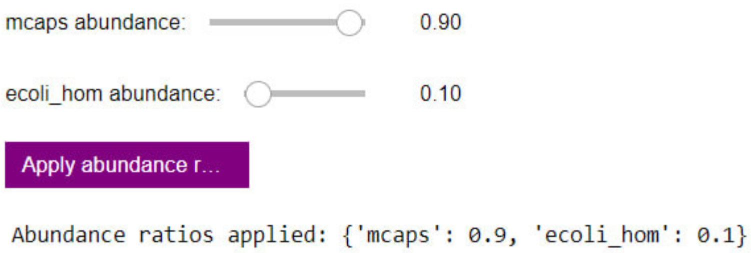

Supplementary Figure S10. A slider for setting the abundance of microorganisms in the community, with the sum required to equal 1.

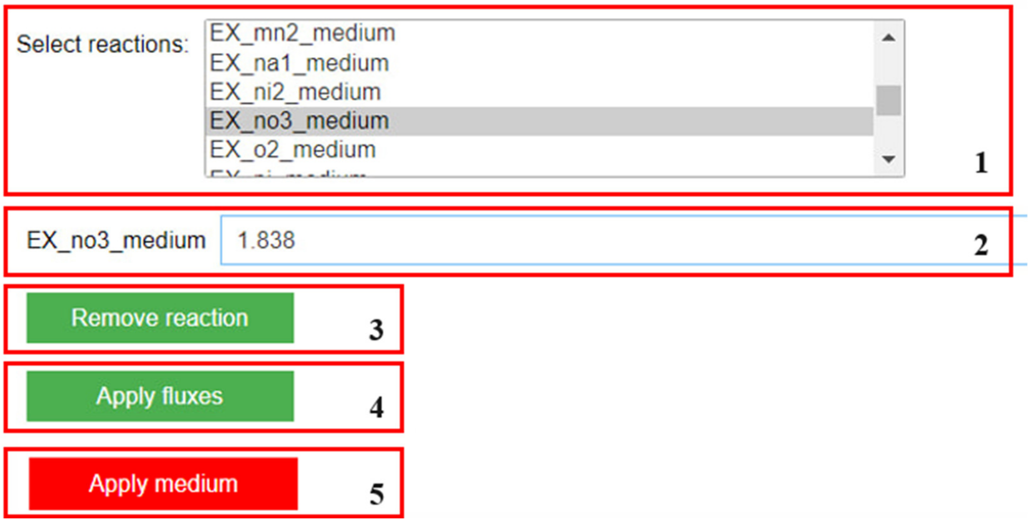

Supplementary Figure S11. The step for set up the environment parameter for the community model consists of: **1** A selector for exchange reactions in the community; **2** A window for setting the boundaries for the chosen reactions; **3** A button to remove a metabolites from the environment; **4** A button for setting a boundary for reactions to continue the modification of metabolites in the environment; **5** A button for adding the configured environment to the community model.

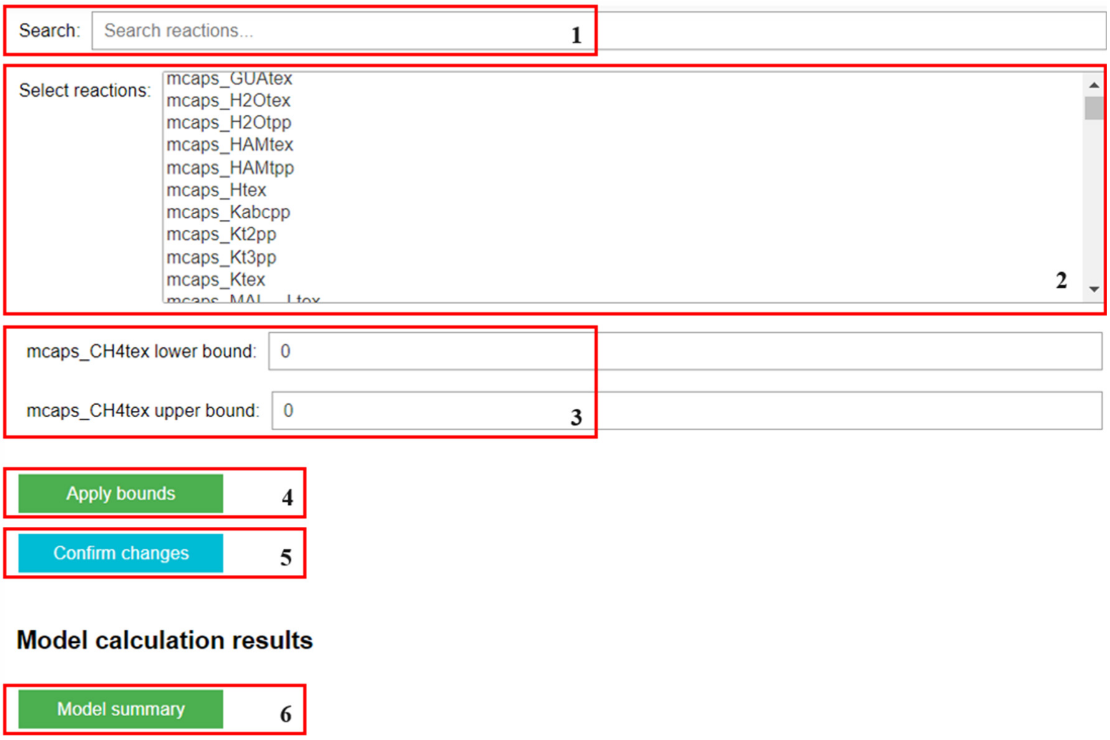

Supplementary Figure S12. The step for modifications of reactions in the community model includes: **1** A window for searching reactions in the community model; **2** A selector to choose a reaction; **3** Changing the upper and lower bounds of the reaction; **4** A button for setting the boundaries of the selected reaction; **5** A button to confirm and apply those modifications, and 6 - a button for displaying the model summary.

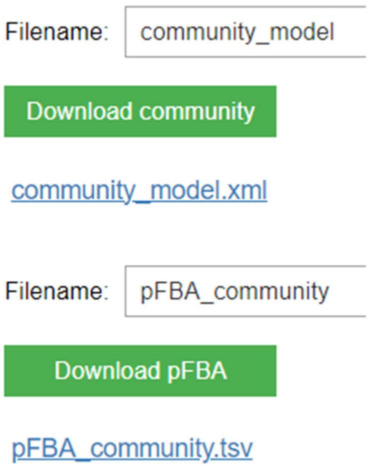

Supplementary Figure S13. The step includes buttons for downloading the built community model in SBML format and pFBA results with all calculated fluxes for the community model.

Supplementary Tables

Supplementary Table S1: Solutions found by OptFlux for the *E. coli* model.

Reaction	Growth rate, h^{-1}	Homoserine production, $mmol*gDCW^{-1}*hr^{-1}$
R_PItex = 8.0<br>R_ICL = 0.5	0.355	5.378
R_PItex = 8.0<br>R_FCLT = 32.0	0.361	5.372
R_PItex = 8.0<br>R_CU2tpp = 16.0	0.369	5.365
R_PItex = 8.0	0.369	5.365

Supplementary Table S2: Cross-metabolites of the *i*McBath and *i*EC1372_W3110 community without homoserine production from *i*EC1372_W3110 and with its production.

Metabolite	Producer	Consumer	o2_lim, $mmol*gDCW^{-1}*hr^{-1}$	o2_lim_homoserine, $mmol*gDCW^{-1}*hr^{-1}$	no3_lim, $mmol*gDCW^{-1}*hr^{-1}$	no3_lim_homoserine, $mmol*gDCW^{-1}*hr^{-1}$
Acetate	*M.capsulatus*	*E.coli*	3.343	0.205	0.612	-

Aspartate	*E.coli*	*M.capsulatus*	0.515	-	-	-
Fumarate	*M.capsulatus*	*E.coli*	-	-	-	0.096
Glycerol	*M.capsulatus*	*E.coli*	-	5.3E-4	3.3E-18	6.2E-4
Glycerol	*E.coli*	*M.capsulatus*	0.908	-	-	-
H_2O	*M.capsulatus*	*E.coli*	30.896	-	-	-
Homoserine	*E.coli*	*M.capsulatus*	0.282	0.575	0.126	0.572
Malate	*M.capsulatus*	*E.coli*	-	1.144	0.361	1.197
Na^+	*E.coli*	*M.capsulatus*	-	0.007	2.2E-20	-
NH_4	*E.coli*	*M.capsulatus*	-	-	0.071	-
NH_4	*M.capsulatus*	*E.coli*	0.981	0.7755	-	0.777
NO_2	*M.capsulatus*	*E.coli*	0.059	-	-	-
Pi	*E.coli*	*M.capsulatus*	0.112	-	0.108	-
Serine	*E.coli*	*M.capsulatus*	0.151	0.467	-	0.445
Succinate	*E.coli*	*M.capsulatus*	4.06E-4	3.3E-4	-	-
Threonine	*M.capsulatus*	*E.coli*	0.151	0.467	-	0.445

Supplementary Table S3: Reactions required for considered electron transfer mechanisms in the *i*McBath model.

Electron transfer mechanism/ Reactions	sMMOi	pMMODCipp	pMMOipp	CyLCyHORpp	NADH16pp	NADHNQR2ipp	NADH5ppi	CYOR_q8ppi
**Redox-arm**	0	-	(0,1000)	-	0	0	-	-
**Direct**	0	(0,1000)	-	0	-	-	-	-
**Uphill**	0	-	(0,1000)	-	-	-	0	(-1000,0)

Supplementary Table S4: FBA results of the *i*McBath model under different electron transfer mechanisms.

Model	Original_model, mmol*gDCW⁻¹* hr⁻¹	Uphill, mmol* gDCW⁻¹*hr⁻¹	Redox-arm, mmol* gDCW⁻¹*hr⁻¹	Direct, mmol* gDCW⁻¹*hr⁻¹
**Ratio O2/CH4**	1,5 (27,81/18,46)	1,24 (22,84/18,46)	1,5 (27,81/18,46)	1,13 (20,92/18,46)
**Biomass**	0,178	0,275	0,178	0,313
**Uptake**				
EX_ca2_e	0,01244	0,01921	0,01244	0,02184
EX_ch4_e	18,46	18,46	18,46	18,46
EX_cl_e	0,03816	0,05895	0,03816	0,067
EX_cobalt2_e	1,03E-05	1,59E-05	1,03E-05	1,80E-05
EX_cu2_e	0,0002689	0,0004154	0,0002689	0,0004721
EX_fe2_e	0,001089	0,001683	0,001089	0,001913
EX_h_e	1,458	2,253	1,458	2,561
EX_k_e	0,03141	0,04853	0,03141	0,05516
EX_mg2_e	0,02197	0,03394	0,02197	0,03858
EX_mn2_e	3,20E-06	4,95E-06	3,20E-06	5,63E-06
EX_na1_e	0,006968	0,01076	0,006968	0,01224
EX_ni2_e	6,05E-06	9,35E-06	6,05E-06	1,06E-05
EX_no3_e	1,459	2,253	1,459	2,561
EX_o2_e	27,81	22,84	27,81	20,92
EX_pi_e	0,09814	0,1516	0,09814	0,1723
EX_so4_e	0,03005	0,04641	0,03005	0,05276
EX_zn2_e	0,000178	0,000275	0,000178	0,0003126
**Secretion**				
DM_4hba_c	5,10E-06	7,88E-06	5,10E-06	8,95E-06
DM_doxopa_c	0,0001895	0,0002928	0,0001895	0,0003328
DM_h2o2_c	0,05718	0,08834	0,05718	0,1004
DM_rdmbzi_c	0,0001895	0,0002928	0,0001895	0,0003327
EX_co2_e	12,47	9,204	12,47	7,94

EX_co_e	5,10E-06	7,88E-06	5,10E-06	8,95E-06
EX_h2o_e	32,96	30,8	32,96	29,97
**EX_ac_e**	**-**	**-**	**-**	**-**

Supplementary Table S5: Modified reactions with new boundaries in the *i*McBath model.

Metabolic pathway	Response ID in the model	Set boundaries
**TCA**	MDH	-1000,1000
	CITL	-1000,1000
	ACONTa	-1000,1000
	ACONTb	-1000,1000
	ACONT	-1000,1000
	SSALx	-1000,0
	SSALy	-1000,0
	SUCCt2_2pp	-1000,1000
	FUM	-1000,1000
	ADSL1i	-1000,1000
	ARGSL	-1000,1000
	ME1	-1000,1000
	FUMt2_2pp	-1000,1000
	MALt2_2pp	-1000,1000
**EMP**	PFK_ppi	0,1000
	PYK	0,1000
**RuMP**	HPS	0,1000
	AH6PI	0,1000
	TKT2	0,1000
	RPI	0,1000
	TKT1	0,1000
	TALA	0,1000

	RPE	0,1000
**ED**	G6PDH2	0,1000
	PGI	-1000,1000
	PGL	0,1000
	EDD	0,1000
	EDA	0,1000
	GND	0,1000

Supplementary Table S6: Impact of changes in EDD pathway flux on EMP reaction fluxes in the *i*McBath model (PFK—phosphofructokinase, PFK3—pyrophosphate-dependent 6-phosphofructokinase, PGM—phosphoglycerate mutase), methane and oxygen consumption, and acetate production.

**ED**	**Biomass, h^{-1}**	**ratio O_2/CH_4, mmol* $gDCW^{-1}$*hr^{-1}**	**PFK, mmol* $gDCW^{-1}$*hr^{-1}**	**PFK3, mmol* $gDCW^{-1}$*hr^{-1}**	**PGM, mmol* $gDCW^{-1}$*hr^{-1}**	**Acetate, mmol* $gDCW^{-1}$*hr^{-1}**
0	0	(-12.515/-8.391)	0	0	0	0
0.503	0.277	(-22.744/-18.46)	2.529	0	1.921	0
1.007	0.275	(-22.824/-18.46)	2.009	0	1.404	0
1.510	0.274	(-22.904/-18.46)	1.488	0	0.887	0
2.014	0.272	(-22.983/-18.46)	0.968	0	0.370	0
2.517	0.270	(-23.089/-18.46)	0.442	0	-0.151	0
3.021	0.268	(-23.224/-18.46)	0	-0.091	-0.678	0
3.524	0.265	(-23.359/-18.46)	0	-0.623	-1.205	0
4.028	0.262	(-23.494/-18.46)	0	-1.155	-1.731	0
4.531	0.260	(-23.630/-18.46)	0	-1.688	-2.258	0
5.035	0.257	(-23.765/-18.46)	0	-2.220	-2.784	0
5.538	0.254	(-23.9/-18.46)	0	-2.753	-3.311	0
6.041	0.246	(-23.95/-18.46)	0	-3.157	-3.697	0.191
6.545	0.188	(-25.082/1.111)	0	-3.377	-3.789	1.111

Supplementary Table S7: Additional modifications of the community model.

Reactions of the community model	Boundaries
ecoli_TP_ecoli_eca4colipa_ecoli_e	0,0
ecoli_TP_colipa_ecoli_e	0,0
ecoli_TP_colipap_ecoli_e	0,0
ecoli_TP_btn_ecoli_e	0,0
ecoli_TP_acolipa_ecoli_e	0,0
ecoli_TP_hacolipa_ecoli_e	0,0
ecoli_TP_o16a4colipa_ecoli_e	0,0
ecoli_TP_o6a4colipa_ecoli_e	0,0
ecoli_TP_lipa_ecoli_e	0,0
ecoli_GPDDA1pp	0,0
ecoli_TP_lipa_cold_ecoli_e	0,0
ecoli_hom_TP_ecoli_hom_eca4colipa_ecoli_hom_e	0,0
ecoli_hom_TP_colipa_ecoli_hom_e	0,0
ecoli_hom_TP_colipap_ecoli_hom_e	0,0
ecoli_hom_TP_btn_ecoli_hom_e	0,0
ecoli_hom_TP_acolipa_ecoli_hom_e	0,0
ecoli_hom_TP_hacolipa_ecoli_hom_e	0,0
ecoli_hom_TP_o16a4colipa_ecoli_hom_e	0,0
ecoli_hom_TP_o6a4colipa_ecoli_hom_e	0,0
ecoli_hom_TP_lipa_ecoli_hom_e	0,0
ecoli_hom_GPDDA1pp	0,0
ecoli_hom_TP_lipa_cold_ecoli_hom_e	0,0
ecoli_TP_h_ecoli_e	-1000,0
mcaps_TP_h_mcaps_e	-1000,0
ecoli_hom_TP_h_ecoli_hom_e	-1000,0
ecoli_TP_co2_ecoli_e	0,1000
ecoli_TP_pser__L_ecoli_e	0,0
ecoli_hom_TP_pser__L_ecoli_hom_e	0,0

ecoli_hom_TP_glyclt_ecoli_hom_e	0,0
